# Turbine Rotor Disk Health Monitoring Assessment Based on Sensor Technology and Spin Tests Data

**DOI:** 10.1155/2013/413587

**Published:** 2013-06-05

**Authors:** Ali Abdul-Aziz, Mark Woike

**Affiliations:** NASA Glenn Research Center, Cleveland, OH 44135, USA

## Abstract

The paper focuses on presenting data obtained from spin test experiments of a turbine engine like rotor disk and assessing their correlation to the development of a structural health monitoring and fault detection system. The data were obtained under various operating conditions such as the rotor disk being artificially induced with and without a notch and rotated at a rotational speed of up to 10,000 rpm under balanced and imbalanced state. The data collected included blade tip clearance, blade tip timing measurements, and shaft displacements. Two different sensor technologies were employed in the testing: microwave and capacitive sensors, respectively. The experimental tests were conducted at the NASA Glenn Research Center's Rotordynamics Laboratory using a high precision spin system. Disk flaw observations and related assessments from the collected data for both sensors are reported and discussed.

## 1. Introduction

The strive to develop a robust health monitoring system to detect rotating engine component malfunctions is among the key areas of interest for engine companies and the associated aviation industry. Typically, health monitoring is performed using sensor systems and other similar means that are capable of functioning under harsh and severe environmental operating conditions. Such systems are to operate without interference with the overall operation of the engine. However, implementation of such technology is highly dependent on many factors and among them setting up specific types of experiments to simulate representative turbine engine conditions and frequent mishaps that the engine encounters during operation. Supportive studies like analytical verification and modeling are equally important in order to verify and complement the experimental findings. Testing under high temperature and wireless technology using durable and effective sensor technologies is also highly desirable. 

Health monitoring is not only confined to sensor technology since there are many other ways of conducting such inspection which are mostly nondestructive evaluation-based approaches. These approaches are widely used in the aviation industry to track engine component performance and durability. They are further used to locate cracks and other anomalies before they become a risk factor that leads to catastrophic failure. Nevertheless, some if not most of these techniques can be both costly and impractical, in particular, when it comes to inspecting complex geometries and large structures [1]. Therefore, the urge for developing systematic, reliable and realistic diagnostic tools to detect damage and monitor the health of key components in the engine, such as rotor disks and turbine blades, is highly in need. It is greatly fundamental to maintaining engine safety, dependability, and life [2].

The NASA Aviation Safety Program under the Vehicle Systems Safety Technology (VSST) project is taking the lead in partnership with the Federal Aviation Administration, Aviation Industry and the Department of Defense [3], to promote the development of these technologies to improve and reduce the fatal aviation accidents and assist safety as a whole. This effort is being carried out at NASA Glenn Research Center through the Optical Instrumentation and NDE branch by conducting controlled spin experiments of turbine engine rotor like test articles to explore various sensing advancements for local and global detection of rotor damage. Comparison of test data for baseline disks without any damage with that of a disk with artificially induced damage, a small crack or a notch, is performed to appraise the findings. Hence, this paper presents experimental results obtained from spin tests of a rotor disk and their association to the development of a structural health monitoring and fault detection system. 

## 2. Technical Approach

**Figure 1:**
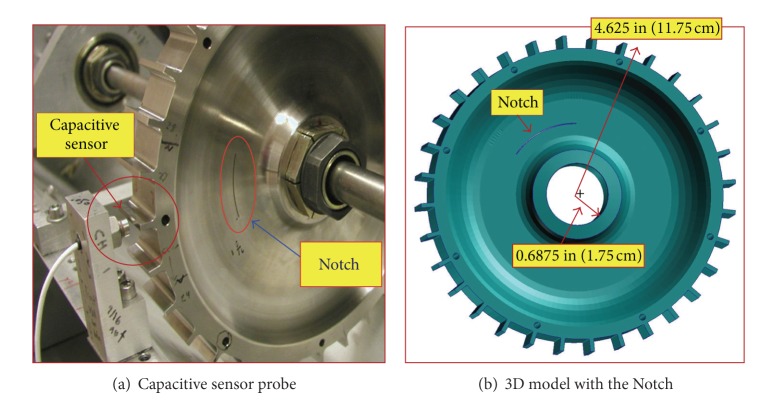
Test disk capacitive sensor assembly.

The experimental work in this study considered a conceptual design of a disk with machined teeth to imitate compressor or turbine blades and provide a cost effective test article to simulate crack initiation and propagation; see Figure 1. The central region of the disk is counter-bored on both sides to create the rim, web, and bore regions of a typical turbine disk. The machined teeth on the rim simulate tip passing, but they trim down the blade mass loading on the web and bore usually experienced in most rotors. The goal is to induce changes in radial tip displacement without disk yielding in order to test the instrumentation and to then initiate and grow cracks by machining and/or increasing rotational speed. Two sensor types (capacitive and microwave) are employed to capture the blade tip clearance both for health monitoring and comparison purposes. An eddy current sensor is also included in the system to measure the shaft displacements. 

Figure 1 illustrates a description of the disk specimen and the induced crack notch along with the tip clearance probes. The test specimen disk has an outside diameter of 23.495 cm (9.25 in), a bore and an outside rim thickness of 2.54 cm (1 in) and 3.175 cm (1.25 in), the thickness of the web is 0.254 cm (0.10 in), and the cross section and height of the blades are 3.175 cm × 0.330 cm (1.25 in × 0.13 in) and 0.838 cm (0.33 in), respectively. It has rotor-like blades, a total of 32, evenly spaced around the circumference. Eight holes, 0.508 cm (0.20 in) diameter each, were drilled through the disk half-way in the rim. The holes were spaced every 45°, and they were designed for future studies as possible mass add-on points or notch initiation sites. The disk specimen is made out of nickel base material alloy Haynes X-750 and it weighs approximately 4.88 Kg (10.75 Lb). 

The notch had a width of 0.381 mm (0.015 in) as per wire thickness and burn area of the electric discharge machining (EDM) process. The notch region was intentionally selected to be in the web area since finite element analysis results revealed that this section encounters the highest stress level in the disk during the spin operation [1, 2]. Technical considerations were emphasized to preserve system consistency of all the operating parameters and other experimental conditions during the removal-reinstallation process of the disk specimen in both situations, baseline no-notch and notch states. 

## 3. Sensor Technology

### 3.1. Capacitive Probe Sensor

For blade tip clearance measurements, a capacitive sensor system was installed; see Figure 1(a). These types of sensors are based on a direct current (DC) offset, an offsetting of a signal from zero where it refers to a direct current voltage, rather than a modulation technique which is a method used to digitally represent sampled analog signals. The capacitive sensors are designed to monitor the electrical property of “capacitance” to initiate and take measurements. The capacitance is a function of the physical dimensions (geometry) of the conductors and the permittivity of the dielectric. It is defined as a field that exists between two conductive surfaces within some rational proximity. Capacitance is directly proportional to the surface area of the conductor plates and inversely proportional to the separation distance between the plates. Variations in the distance between the surfaces lead to changes in the capacitance rate. This rate change is used by the sensors to indicate the difference in position of a target. High-performance displacement sensors use small sensing surfaces and as a result are positioned close to the targets (0.25–2 mm). The DC voltage, in conjunction with the motion of the rotor, allowed the current system to record three channels at a rate of 1 MHz each.

### 3.2. Microwave Sensor Background and Theory

**Figure 2:**
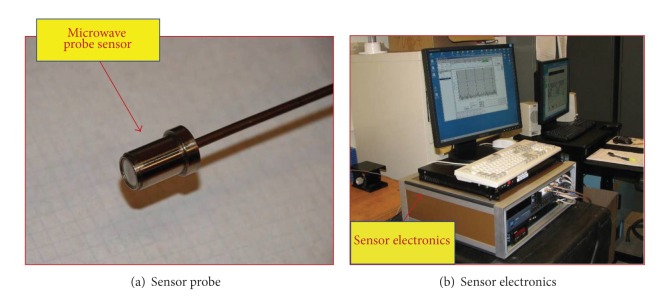
Microwave sensor probe and its electronics setup.

The microwave tip clearance sensor system works on principles that are similar to a short-range radar system. The tip clearance probe is both a transmitting and receiving antenna [3–10]. The sensor emits a continuous microwave signal and measures the signal that is reflected off a passing blade. The motion of the blade modulates the reflected signal. The reflected signal is then compared to an internal reference signal and the phase difference directly corresponds to the distance to the blade. The system consists of two major components. The first component is the probe, (Figure 2(a)). The second component is the sensor electronics, (Figure 2(b)). The probe contains the transmitting and receiving antenna and is designed to be installed in the casing of the engine where it can measure the radial clearance between the face of the sensor and the turbine blade tips. The probes are made of high temperature material and are designed to operate in temperatures of 900°C uncooled, 1200°C with cooling air. Two generations of probes are in operation. The first-generation probes operate at 5.8 GHz and can measure clearance distances up to ~25 mm (i.e., one-half the radiating wavelength). The second-generation probes operate at 24 GHz and in theory can measure clearance distances up to ~6 mm. In regards to physical size, the first generation probes are approximately 14 mm in diameter and 26 mm long. The second-generation probes are approximately 9 mm in diameter and 19 mm long. This technology has an ultimate goal of obtaining clearance accuracies approaching 25 *μ*m. Accuracies in this order were observed in the laboratory during testing [4]. A frequency response of up to 5 MHz is typical, with up to 25 MHz being possible with this technology.

The sensor electronics consist of the radio frequency (RF) generator, RF detector, and all of the associated hardware required to generate, measure, and convert the microwave signals into a displacement reading [4]. 

The sensor electronics are designed to be located off-board of the engine in an environmentally benign area. The probes are connected to the sensor electronics using a microwave rated coaxial cable. A rack-mounted PC is used to interface to the sensor electronics and run the data acquisition and display software. The data acquisition computer is connected to the sensor electronics through a network switch. The data acquisition computer is intended to be remotely located away from the sensor electronics in an area such as a control room using a CAT5E connection.

### 3.3. Capacitive and Microwave Sensors Performance

**Figure 3:**
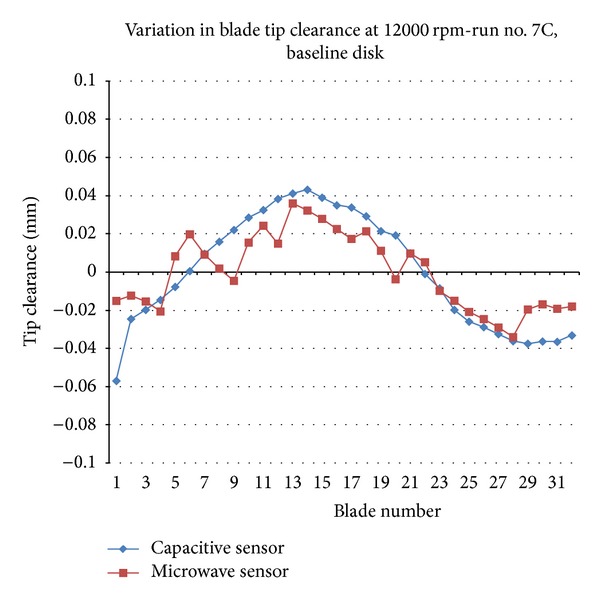
Sample of capacitive and microwave sensor data readings for a baseline disk at 12000 rpm.

Figure 3 demonstrates a comparison between the two primary blade tip clearance sensors, microwave and capacitive, attached to the data system. The data collected for both sensors compared relatively well. However, the similarity is not as uniform, the similarity is not as uniform as anticipated which implies that certain calibration is needed to fine-tune the sensor system further and bring the data closer. Perhaps, adding an additional filtering or average process for the microwave sensor may result in improving the agreement between the two measurements. Nevertheless, each sensor system is operating as expected. Their role in the experiment configuration is to serve the same functionality in a different fashion and to test their performance. 

## 4. Experimental Results

**Figure 4:**
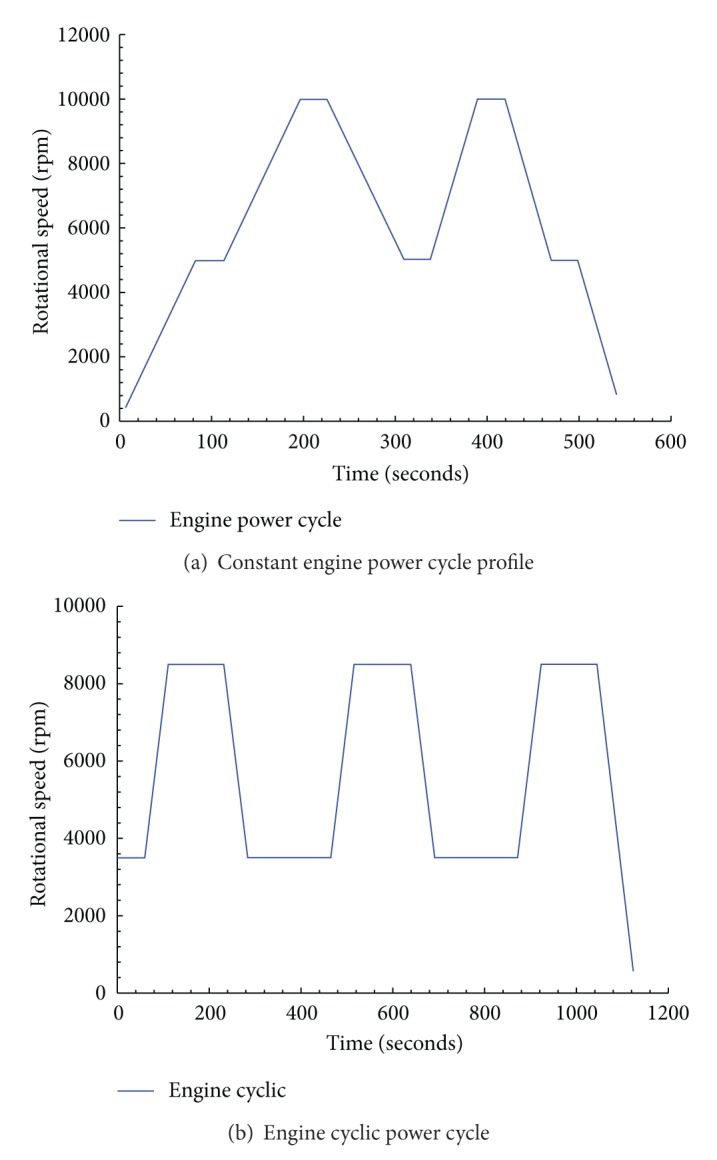
Simulated engine mission history test profiles.

Spin tests were performed on the rotor disk and covered baseline runs with both undamaged and damaged disks via the artificially induced notch, shown in Figure 1. The tests included spinning the rotor under various simulated engine mission profiles starting from a minimum rotational speed of 3000 up to a maximum of 10000 rpm. The controlled speed applied during the current testing was made with an acceleration-deceleration rate of 60 rpm/second. This insured passing the critical speed of 2,610 rpm and leading to postcritical state [11].  Figure 4 shows samples of two mission profiles that were used to test the rotors. These profiles were derived on the basis of revolutions per minute data obtained on different flights comprising different flight maneuvers [12]. 

Figure 4(a) is referred to as the constant engine power cycle profile; however the graph shown does not illustrate the constant behavior due to an input offset. The engine speed reaches 10,000 rpm in two steps, a take off with a brief hold up at 5000 rpm and 40 seconds hold at 10,000 rpm with a rapid decrease to 5000 rpm and a ramp up to 10,000 rpm for one repetitive cycle.  Figure 4(b) shows another mission profile (engine cyclic power cycle) that allows the rotor disk to go through somewhat analogous series of events starting at a speed beyond the critical value [11] and up to 8500 rpm. These profiles are being used to imitate unusual engine conditions and to help in evaluating the rotor performance under harsh and complex loading events in an attempt to fatigue the disk with the expectation that all the existing anomalies would appear in the test data. Additionally, under these conditions, these experiments supplied valuable assessments for both the crack detection scheme and the structural durability of the disk materials.

### 4.1. Spin Test Results

**Figure 5:**
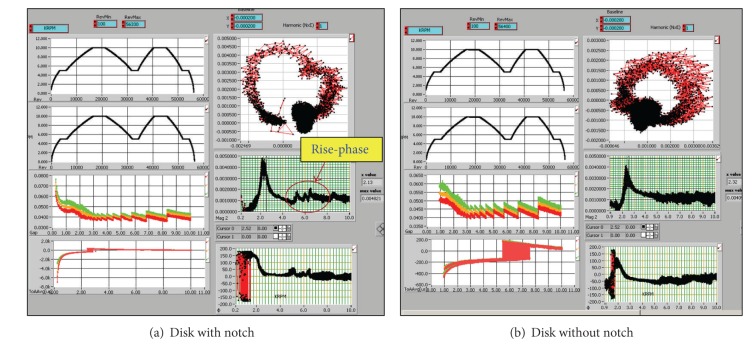
Bode Plots 9 minutes comparison test of the disk with and without notch, capacitive sensor data, Engine cyclic power profile at 5–10 Krpm rotational speed.

**Figure 6:**
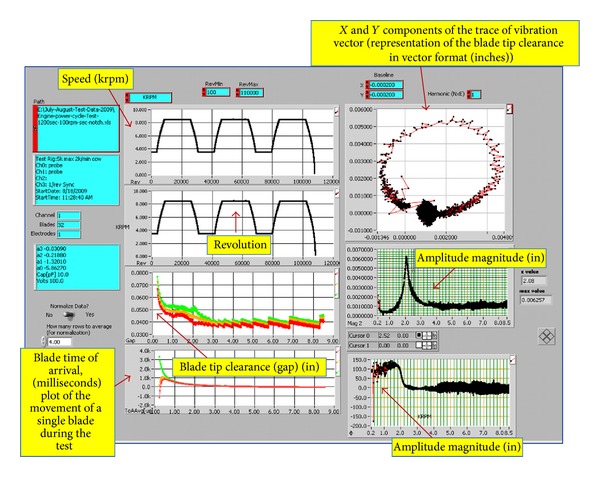
Engine constant power cycle test: capacitive sensor data at 8500 rpm, 1200 sec long at 100 rpm acceleration/deceleration rate for the Notched disk.

Experimental data under both mission profiles are represented in Figures 5 and 6. The data in Figure 5 are produced under the constant power cycle mission for both the baseline no-notch and the notched disks. The mission history is shown along with the trace vibration vector and Bode plots for the phase and amplitude response. Bode plots are a very useful way to represent the gain and phase of a system as a function of frequency. This is referred to as the frequency domain behavior of a system. The magnitude and phase plots determine the phasor representation of the transfer function at any frequency. It is typically used for transient analysis in both run-up and run-down tests. It can help identify the resonance speed of a rotor or examine the rotor dynamics on an order basis. The *x*-axis in a Bode plot is speed or frequency, which enables seeing the changes in magnitude and phase over speed or frequency.

A clear observation of data variation is noted in Figures 5 and 6 between the two plots. For instance, a circular loop representation for the trace of vibration vector (disk vibration response) is seen for the baseline disk, while a gap in the loop is present for the notched disk. This behavior hints that a difference in the vibration response for the two structures is present signifying the existence of some type of irregularity. Such observation has been reported in [13–17], where a crack in the rotor disk is documented via the presence of distorted trace of the vibration vector distribution and a rise in the phase and amplitude response upon surpassing the 1st critical speed; *see Figure 6 for additional clarifications and captions notation, axes labels and units.*

**Figure 7:**
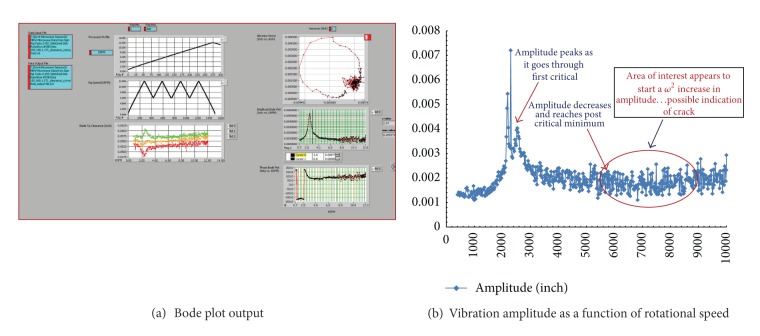
Simulated engine mission history profile (constant amplitude) test, microwave sensor data.

This conduct is certainly noticeable in Figure 5(a) for the notched disk. A rise in the phase magnitude response is noted. Also, it is noted that the peak is at a critical frequency (5000–9000 rpm), and then it begins to settle out at maximum speed close to critical frequency. So the damping ratio keeps the curve from flattening compared to no crack, (Figure 5(b)). Note how the magnitude phase graphs no longer represent a complete circle in the notched disk case, (Figure 7(a)).

This is a sign of a crack growing and is detected from the plot of a cycle worth of data. At the same time the magnitude graph (Figure 5(a)) has started a *ω* ^2^ (*ω* is the rotational speed) rise after settling past the critical frequency. A rise in amplitude and constant phase is typically an indication of the growth of a crack, assuming that some internal movement of the rotating structure does not cause the unbalance [13]. In this case the system is tracking a crack growing in the disk. Therefore, examination of the above data has verified that the detection scheme based on the blade tip clearance response allows identifying the presence of some sort of fault in the rotor disk. However, further confirmation is warranted throughconducting more tests for different rotors under similar operating conditions to authenticate that this type of behavior in the vibration response is accurately due to some existing structural defects in the rotor rather than a system-related unbalance [18–20]. 

Figure 6 shows the results obtained under the cyclic run time mission profile. The trace of vibrations vector in the Bode plot contains rather distorted data with an incomplete circular shape. Also, the amplitude shows a mild rise at the 5000–6000 rpm range which substantiates the theory of the presence of an unbalance condition or an anomaly state as noted in the data presented in Figure 5. However, the manifestation of such observations remains not as straightforward but it underlines or it confirms the presence of some type of defect. Still, further work to confirm this scrutiny is needed.

The data reported in Figure 7 shows the test output of the microwave sensor for a constant amplitude engine history profile. Only data for the takeoff portion of the profile up to the first 300 revolutions is shown, (Figure 7(a)). This is for a 1500 seconds long test at 100 rpm acceleration/deceleration rate for a notched disk. The response of the microwave sensor is very similar to that of the capacitive sensor; it has been introduced into the testing scheme to investigate its applicability and performance for engine health monitoring applications. And as mentioned earlier, the microwave tip clearance sensor system works on principles that are similar to a short-range radar system and are different than those for the capacitive sensor. The probe is both a transmitting and a receiving antenna; it emits a continuous microwave signal and measures the signal that is reflected off a rotating blade.

Figure 7(b) shows a magnified phase amplitude output produced by the data obtained from the microwave sensor for a spin test of a notched disk at 10,000 rpm rotational speed. It is obviously noted that a rise in the phase exhibiting a second-degree order (*ω* ^2^) is recognized at a speed range of 7000 to 10000 rpm. This supports the observations made earlier for the capacitive sensor data concerning the crack detection phenomena in the rotor and the similarity of the microwave sensor performance; see Figure 5. 

### 4.2. Unbalance Test Results

**Figure 8:**
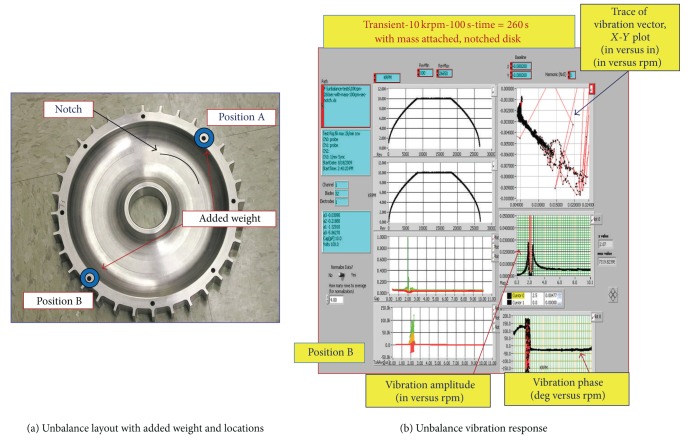
Tip clearance variation with blade numbers and simulated rotor unbalance layout.

An unbalance test was performed to institute a baseline database for the rotor at various operating conditions and to support investigating and evaluating the vibration response under nonordinary service environment such as imbalance situations. The test was conducted at 10,000 rpm at 100 seconds acceleration/deceleration rate and lasted 4.33 minutes long. The test covered a standard mission profile under transient ramp up, cruise, and ramp down conditions. Test conditions were kept the same as those applied during the non-unbalance state to enable precise assessments of the imbalance factors and their impact on the rotor vibrations response under consistent and refined test margins for the same rotor. Figure 8(a) shows a photo of the disk used for the unbalance tests with labels indicating the sites of the locations of the weight (0.5 gram) during the tests. 

As noted, two cases were considered: one case with the extra weight being along the notch side (position A) and another one with weight being across from the notch (position B). Additionally, the purpose of the unbalance test is to check the capability of the sensors technology not only in crack detection, but also in predicting other major malfunctions in the rotor system such as unbalance provision. This further supports the theory which relates to rotor design; whereas the rotational velocity of any rotating object increases, its level of vibration often passes through a maximum at what is called a critical speed [11]. This is commonly excited by unbalance of the rotating structure. If the amplitude of vibration at these critical speeds is excessive, catastrophic failure can occur. For this reason, it is typically recommended that in large rotors design the appropriate approach is to use physical prototypes and tests in order to ensure safe rotating machinery design and balance it well.

Figure 8(b) shows a test that runs for the notched rotor disk under unbalance test with the mass being at position B as indicated in Figure 8(a). Position B is on the opposite side of the notch. This location was intentionally chosen to investigate imbalance effects not only at positions within the region surrounding the notch, but also at areas outside the notch section and further out. Test results at “position A” are not shown due to space limitation and since similar results were observed as well.

It is also noted that the trace of vibration vector shown in Figure 8(b) shows a very irregular distribution signifying that the imbalance had eliminated the typical complete circular shape usually encountered when a clean baseline rotor disk without any balance weight or damage is in operation. This further designates that having an imbalance state whether it is due to the existence of a notch or other factors within the system will lead to nonordinary shape of the trace of the vibration vector curve. Additionally, these data confirm that upon altering the disk weight by adding or removing mass, the state of imbalance is imminent and the tip clearance magnitude will depend on the added mass size and location. Further, the findings of these results substantiated the purpose of these tests to help determine that the blade tip clearance sensors have recognized the significance of these critical parameters and their influence on the disk performance. 

## 5. Conclusions

The work performed in this paper involved conducting spin experiments on the rotor disk with and without an artificially induced notch at different rotational speed levels. Bode plots of data from these tests provided indications of differences between the undamaged rotor with that of the damaged one. Crack growing signs have been detected where the amplitude magnitude in the Bode plot has started a *ω* ^2^ rise after settling past the critical frequency. This growth of the magnitude is a noteworthy indicator of a crack growth or another fault which confirms similar results cited by prior studies. Microwave and capacitive sensors delivered closely similar data for most of the blades except for some where the measurements were slightly dissimilar which can be attributed to factors such as equipment calibration and apparatus instrumentation fine tuning.

The trace of vibration vector which represents the imbalance mass showed very inconsistent distribution compared to data obtained from a balanced disk. This showed that the imbalance had eliminated the complete circular shape typically encountered when a clean baseline rotor disk without any damage is in operation. Additionally, this indicated that having an imbalance state whether it is due to the existence of a notch or other factors within the system, the trace of the vibration vector curve will have an asymmetrical non-circular and highly distorted shape. This leads to the conclusion that the data obtained from spin testing of the rotor to some extent showed that the detection scheme based on the blade tip clearance response is capable of identifying the presence of defects in the rotor.

Lastly, the experimental data enabled exploring the difference in the vibration response between a baseline and a damaged rotor suggesting when the existence of some type of anomaly is present. Also, the combined sensor technology, which included the capacitive, microwave and eddy current probes, supplemented the tests with ample evidence and allowed exploring the changes in the disk vibration response at different operating conditions. However, further work and more testing must be continued to develop, improve and link this experimental investigation to put forward a more precise and accurate appraisal of monitoring the health of rotating components.
